# Unravelling the structures of sodiated β-cyclodextrin and its fragments†

**DOI:** 10.1039/d1cp01058a

**Published:** 2021-06-07

**Authors:** Jordan M. Rabus, Robert P. Pellegrinelli, Ali Hassan Abi Khodr, Benjamin J. Bythell, Thomas R. Rizzo, Eduardo Carrascosa

**Affiliations:** Department of Chemistry and Biochemistry, Ohio University 391 Clippinger Laboratories Athens Ohio 45701 USA; Laboratoire de Chimie Physique Moléculaire, École Polytechnique Fédérale de Lausanne, EPFL SB ISIC LCPM Station 6 CH-1015 Lausanne Switzerland eduardo.carrascosacasado@epfl.ch

## Abstract

We present cryogenic infrared spectra of sodiated β-cyclodextrin [β-CD + Na]^+^, a common cyclic oligosaccharide, and its main dissociation products upon collision-induced dissociation (CID). We characterize the parent ions using high-resolution ion mobility spectrometry and cryogenic infrared action spectroscopy, while the fragments are characterized by their mass and cryogenic infrared spectra. We observe sodium-cationized fragments that differ in mass by 162 u, corresponding to B_*n*_/Z_*m*_ ions. For the *m*/*z* 347 product ion, electronic structure calculations are consistent with formation of the lowest energy 2-ketone B_2_ ion structure. For the *m*/*z* 509 product ion, both the calculated 2-ketone B_3_ and the Z_3_ structures show similarities with the experimental spectrum. The theoretical structure most consistent with the spectrum of the *m*/*z* 671 ions is a slightly higher energy 2-ketone B_4_ structure. Overall, the data suggest a consistent formation mechanism for all the observed fragments.

## Abstract 2

Experiment and theory unravel the structure and formation mechanism of β-cyclodextrin fragment ions.

## Graphical abstract

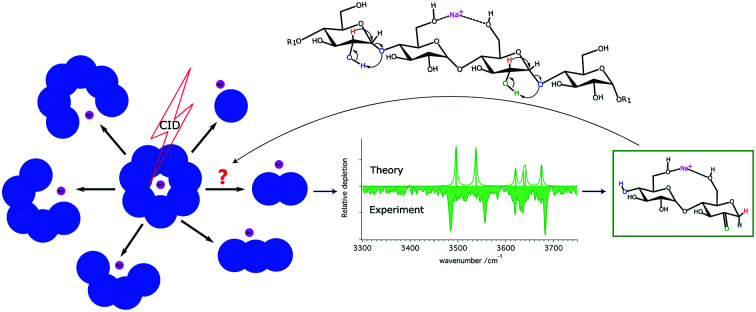

## Introduction

The synthesis and biomedical applications of large cyclic molecular systems have received growing attention, mainly due to their ability to bind selectively to substrates based on non-covalent interactions.^1^ Crown ethers are arguably the most popular and best investigated host molecules for complexing metal ions and are widely used in organic and supramolecular chemistry.^2,3^ Cyclic oligosaccharides occur both naturally and synthetically, and they share similar properties with crown ethers in being able to act as a host for metal cations, organometallic complexes, and biologically relevant molecules.^4^ Among the advantages of these compounds is their relative ease of derivatization, allowing for a suitable functional group to be attached to the cyclic oligosaccharide, which can alter their affinity to a certain enantiomer and thus make them good chiral selectors. This is of great interest in separation techniques such as liquid chromatography and capillary electrophoreses.^5,6^

The best-known cyclic oligosaccharides are cyclodextrins (CDs), which are produced enzymatically from starch and occur in three different configurations: α, β, and γ, made up of 6, 7, and 8 d-glucopyranose units, respectively, and linked together by α-(1-4) glycosidic bonds.^7^ The non-toxicity and inexpensive synthesis of cyclodextrins has made them the molecules of choice for many applications including drug delivery, cosmetics, and food processing.^8–11^ Very recently, a modified β-CD has been shown to have broad spectrum anti-viral properties, including against herpes and Zika.^12^

**Fig. 1:**
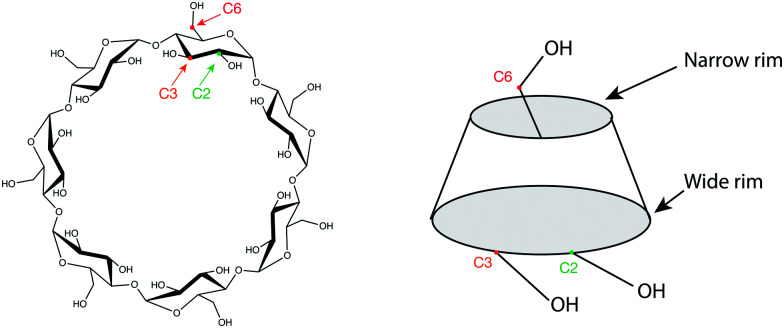
Schematic representation of β-cyclodextrin, with its conical structure represented on the right. The carbon atoms connected to the hydroxyls are labelled C2, C3 and C6 for one glucose unit.

The properties of cyclodextrins arise from their unique conical cylindrical structure, with the hydroxyls pointing outwards while the glycosidic oxygens and the hydrogens line the interior,^13^ leading to a hydrophobic zone inside the cylinder, and a hydrophilic zone on the exterior. This allows CDs to form complexes with a wide variety of compounds, ranging from nonpolar molecules that are captured in the hydrophobic cavity, to polar molecules and ions which preferentially interact with the outer polar surface. Because of this feature, cyclodextrins are water soluble and can be used to bring poorly soluble compounds into aqueous solution by enclosing them in their hydrophobic cavity. For β-CD, the C2 and C3 hydroxyls form a complete hydrogen-bonded network on the wide rim, while the C6 hydroxyls form a separate network on the narrow rim, resulting in a rigid structure (Fig. 1).^14^

Complexation of CDs with guest molecules and ions can distort these structures. It is therefore important to identify these subtle structural details and determine how they affect the physicochemical and biological properties of these species. For this purpose, techniques are needed that can isolate, control and probe molecules. Tandem mass spectrometry (MS^*n*^) coupled with soft-ionization techniques, such as electrospray ionization (ESI) and matrix-assisted laser desorption (MALDI), has emerged as a key tool to investigate large molecules and complexes.^15^ Mass spectrometry has thus been used to study many properties of cyclodextrins, including their host–guest interaction with various organic molecules, their binding selectivity with different metal cations, and their fragmentation patterns in the gas phase.^16–21^ MS coupled to collision-induced dissociation (CID) is often used in biomedical and pharmaceutical contexts to reconstruct the primary structure and binding characteristics of complexed and functionalized CDs. For instance, fragmentation spectra can help determine the chirality of target analytes present in CD-based host–guest complexes,^22,23^ as well as identify regioisomers of functionalized CDs.^20^ Furthermore, studies that use CDs to promote or catalyze the synthesis of biodegradable products rely on CID-MS studies to determine the number and structural arrangement of derivative host units attached to the CD guest, and consequently optimize the synthetic strategy.^24,25^ The homomolecular nature of cyclodextrins makes them interesting models for CID studies, because they are limited to three types of fragmentation: B/Y, C/Z and cross-ring fragmentation.^26^ Fragmentation of β-CD–metal cation complexes by CID has primarily shown a series of product cations separated by 162 u, which corresponds to a dehydrated glucose unit that requires fragmentation of two glycosidic linkages.^26–28^ In addition, other cross-ring fragmentation channels have been observed, especially for anions.^29^

Despite being a powerful tool for fragmentation analysis, MS alone is unable to provide all desirable isomer-specific information, such as preferential isomer formation upon reaction or fragmentation. In the case of cationized cyclodextrins, MS studies have been unable to address whether CID preferentially forms B_*n*_ or Z_*m*_ ions. However, the addition of a vibrational spectroscopy dimension to MS can help identify structural details of CID fragments. While vibrational spectroscopy of ions in mass spectrometers dates back to early ‘80's from the work of Beauchamp and coworkers,^30,31^ the development of user-friendly infrared lasers has resulted in the reemergence of infrared (IR) action spectroscopy as a structural probe for gas-phase ions, including biomolecular ions.^32–35^ Our group has recently demonstrated that cryogenic IR spectroscopy coupled with ion mobility spectrometry (IMS) is able to identify various isomeric glycans ranging from monosaccharides to nine-membered N-glycans.^36–39^ In a very recent study we have been able to identify anomeric CID fragments from single glycans by their IR spectra.^40^ The advantage of measuring spectra of cryogenically cooled ions is that it greatly reduces thermal inhomogeneous broadening, resulting in significantly enhanced spectral resolution, which is particularly important for the analysis of large molecules possessing inherently congested spectra.^33^

Here we investigate the CID products of sodiated β-CD using cryogenic IR spectroscopy. Understanding which fragmentation pathway(s) is (are) responsible for product ions will provide insight into potential dissociation mechanisms of oligosaccharides which, until now, remain only partially understood.

## Methods

### Experimental details

**Fig. 2:**
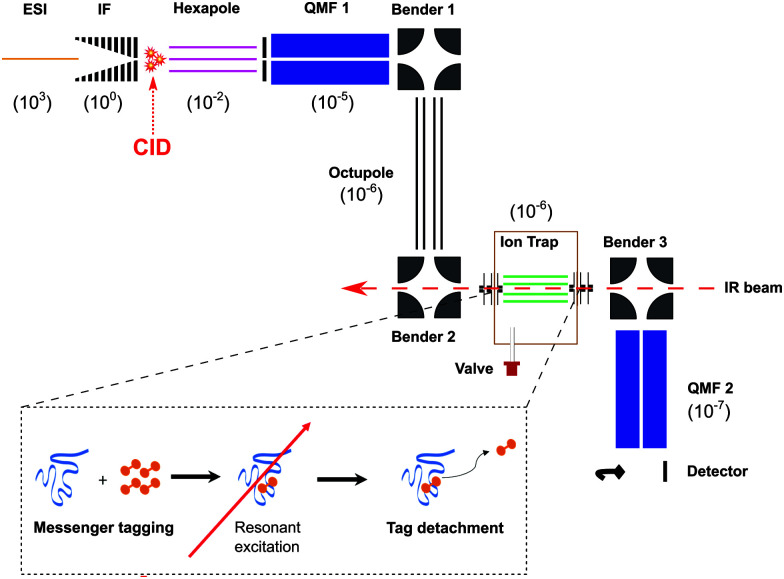
Schematic representation of the experimental setup to obtain IR spectra of mass selected CID fragments under cryogenic conditions. Pressures, in mbar, are indicated in brackets for each differentially pumped section. The bottom left inset illustrates the principle of messenger tagging spectroscopy.

To explore the fragmentation characteristics and corresponding structural signatures of [β-CD + Na]^+^, tandem mass spectrometry, collision-induced dissociation and laser spectroscopy are combined in a homebuilt instrument, schematically depicted in Fig. 2, the details of which have been previously reported.^41^

β-Cyclodextrin (purchased from Sigma Aldrich and used with no further purification) was dissolved in a water–methanol (1 : 1) mixture at 0.1 mM concentration, complexed to group I cations, and introduced into the gas phase *via* nano-electrospray (nESI). In the case of sodiated β-CD cations, the electrosprayed ions were radially confined in an RF ion funnel at ∼1 mbar before being accelerated through a potential difference of ∼240 V into a hexapole ion trap at a pressure of 10^−2^ mbar, creating fragments *via* CID. The trapped precursor ions and their fragments were then extracted as 100 μs pulses at 10 Hz and passed through a quadrupole mass filter, where ions of a designated *m*/*z* range are selected. The transmitted ions were subsequently guided through two electrostatic benders and an octupole ion guide before being focused into a linear octupole ion trap that is cooled by a closed cycle cryocooler (SHI Cryogenics). Upon entering the trap, the ions are thermalized through collisions with a He/N_2_ buffer gas mixture (90 : 10) that was previously introduced as 250–350 μs pulses and pre-cooled to the temperature of the copper trap housing. The trap temperature was held at 50–60 K using a resistive heater attached to the copper housing to avoid condensation of N_2_. To obtain single-photon infrared spectra at cryogenic conditions, the target ions were “tagged” by forming weakly bound complexes with N_2_. The trapped messenger-tagged species were then irradiated with IR light from a pulsed tunable optical parametric oscillator (OPO, LaserVision). In the event of resonant absorption, the vibrational energy rapidly redistributes within the molecule, thereby detaching the weakly-bound N_2_ tag. All the ions were then extracted from the trap and passed through a second quadrupole mass filter where they were detected using a Channeltron. By selecting the *m*/*z* associated with the messenger-tagged target ion and monitoring its depletion as a function of laser wavenumber in a laser on–off experiment, cryogenic infrared action spectra were obtained. These spectra were normalized to the OPO power, which slightly varies over the wavelength range of the scan.

To investigate whether multiple isomers coexist in the gas phase for the studied species, analogous solutions of sodiated β-CD and α-CD were electrosprayed into a recently developed ultrahigh-resolution ion mobility spectrometer attached to a cryogenic trap and a time-of-flight mass spectrometer.^42^ The ion mobility device allows for separation path lengths of over 10 meters and has demonstrated the ability to separate structurally analogous saccharide anomers. Thus, structural differences arising from differing hydrogen bonding arrangements, ring deformations (chair, boat, skew), and sites of sodiation should be well resolved.

### Computational approach

To understand the formation mechanism and structure of the observed fragments, theoretical simulations were carried out on the three smallest fragment ions. As previously,^43–46^ simulations were performed using the genetic algorithm tool Fafoom^47,48^ to enable effective characterization of the potential energy surface. The structures were optimized using the MMFF94 force field.^49–53^ This approach samples a wide range of ring structures incorporating multiple chair, boat, and skew forms, enabling a thorough interrogation of the potential energy surface. All oxygen sites had sodium cations added using custom scripts to generate starting points for the calculations. Geometry optimizations of the resulting candidate conformations were performed with the Gaussian 09 software package^54^ at the HF/3-21G, B3LYP/6-31G(d), and B3LYP/6-31+G(d,p) levels of theory.^55–57^ For the putative charged fragments of the largest species with *m*/*z* 671, a combination of a preliminary PM6 optimization with a HF/3-21g single point energy calculation was performed prior to the aforementioned series of calculations. Degenerate structures were removed at each stage, and only the most competitive non-degenerate structures were optimized at each successively more realistic level of theory. Additional, targeted manual adjustment and supplementation of the structural pool analysed were performed to reduce the chance that chemically relevant species had been neglected. Subsequent simulations utilizing the CREST package,^58–60^ which systemically places a sodium ion at each lone pair of the molecule, with subsequent molecular dynamics using a semiempirical engine followed by B3LYP/6-31G(d) and B3LYP/6-31+G(d,p) calculations broadened the pool of low (and high) energy candidate structures but failed to generate new global minima. All minima were tested by vibrational analysis (all real frequencies). The potential energy surface generated combined the zero-point energy correction (ZPE) to the electronic energy (*E*_el_, 0 K) for improved accuracy (Δ*E*_el+ZPE,0K_). The related, standard enthalpy (Δ*H*_298K_), Gibbs free energy (Δ*G*_298K_), and entropy (Δ*S*_298K_) corrections to 298 K were also determined. Calculated B3LYP/6-31+(d,p) vibrational frequencies were utilized for comparisons with the experimental spectra. A scaling factor of 0.955 was used for all vibrational frequencies and a 5 cm^−1^ full width at half maximum Lorentzian line shape was employed for comparison to the experimental spectra. Targeted single point calculations at the M06-2X, and ωb97X-D levels of theory were also performed on the selected minimum energy structures to help address variability. Subsequent targeted M06-2X and ωb97X-D optimizations and frequency calculations with 6-31+G(d,p) basis sets on *m*/*z* 671 candidates were consistent with the B3LYP data.

## Results and discussion

### CID mass spectrum

**Fig. 3:**
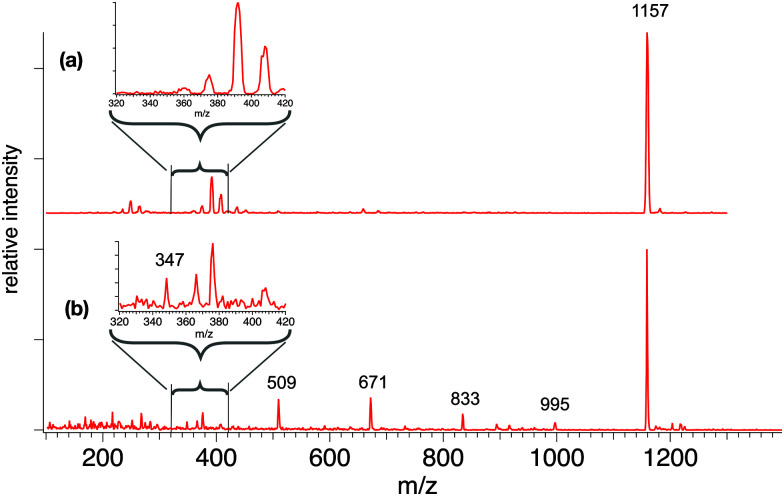
CID-MS of sodiated β-CD and its fragments, carried out with a potential difference of 100 V (a) and 240 V (b) in our tandem mass spectrometer. Consecutive loss of 162 *m*/*z* is observed from the parent at 240 V, while no fragments are observed at 100 V.

Fig. 3 presents mass spectra of sodiated β-CD under largely non-dissociative conditions (a) and CID conditions (b). The spectrum in Fig. 3(b) exhibits the [β-CD + Na]^+^ precursor ion mass at *m*/*z* 1157, as well as a sequence of lower mass peaks at *m*/*z* 995, 833, 671, 509 and 347.

This series indicates spacings of 162 u, a fragment moiety that has been previously observed^20,27^ and corresponds to a dehydrated glucose monosaccharide. Detection and analysis of the sodiated monomeric dehydrated glucose fragment at *m*/*z* 185 was not attempted, mainly due to the increased number of overlapping ions in the low mass range. A minor peak at *m*/*z* 893 corresponds to the loss of 264 mass units and is ascribed to a minor cross-ring fragmentation process also observed in previous investigations.^27,29^ The masses corresponding to the three- and four-glucose unit fragments (*m*/*z* 509 and *m*/*z* 671) appear as the most intense CID products, whereas formation of two- and six-residue fragments appear with much lower intensity.

It is difficult to determine whether the fragmentation occurs in a sequential or simultaneous manner. However, similar peaks and relative intensities are produced in a commercial time-of-flight (ToF) spectrometer (Waters Q-Tof Premier, Fig. S1, ESI†) and our home-built instrument. To further investigate this, a series of CID experiments at increasing collision energies were performed on the Q-Tof, and all the main fragments appeared at the same energy threshold, providing no evidence for a sequential process (see Fig. S2, ESI†).

### Ion mobility and cryogenic IR action spectra of sodiated α-CD and β-CD

**Fig. 4:**
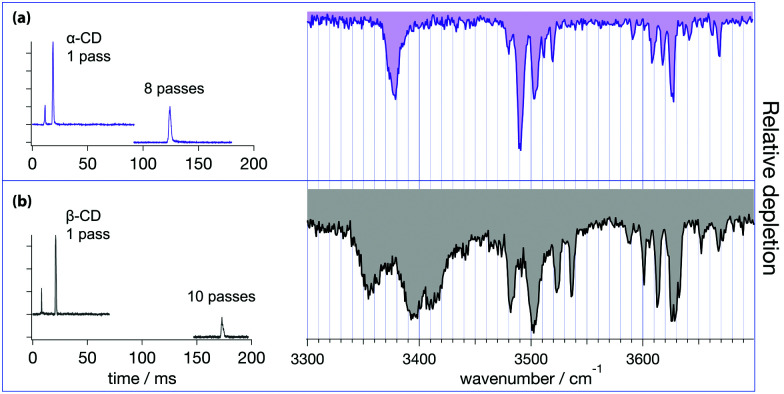
Arrival times of [α-CD + Na]^+^ (a) and [β-CD + Na]^+^ (b) and their corresponding cryogenic IR spectra. Note that the fast ATD peak in the left panels of (a) and (b) corresponds to doubly charged dimers of the corresponding cyclodextrins that cannot be discriminated by our quadrupole mass filter and are propelled faster through the IMS buffer gas due to their doubly charged character.

Precursor [β-CD + Na]^+^ ions can adopt different conformations depending on the relative orientation of the OH groups at both the narrow and wide rims of the conical cylindrical structure (Fig. 1). The relative orientation of these donor–acceptor hydrogen bonding networks at both edges of the molecule can potentially lead to four different combinations, depending whether the hydrogen bonding pattern is clockwise (cw) or counter-clockwise (cc): cc–cc, cc–cw, cw–cc and cw–cw.^61^ Distortion of these symmetric conformations through inclusion of Na^+^ might significantly increase the number of conformers. To evaluate whether electrosprayed [β-CD + Na]^+^ ions form a distribution of conformers, additional experiments were carried out using a recently designed instrument that combines ultrahigh-resolution travelling-wave ion mobility spectrometry (IMS) using structures for lossless ion manipulation (SLIM),^62,63^ with cryogenic infrared spectroscopy to provide isomer-specific structural identification of complex molecules.^42^ A schematic representation of this instrument is shown in Fig. S3 (ESI†). In this work, sodiated β-CD was cycled through the IMS section of this apparatus over a total length of ∼17 meters, which has been shown to be sufficient to separate small structural differences.^39^ However as shown in Fig. 4(b), only one primary, well-resolved peak was observed in the arrival time distribution (ATD) under these conditions. A similar experiment on the six-member cyclic structure [α-CD + Na]^+^ also reveals a single peak in the ATD (Fig. 4(a)). These results can be potentially interpreted in three ways: (1) the monomodal ATDs demonstrate the presence of a single, stable gas-phase conformer for both sodiated α- and β-CD; (2) both cyclodextrins consist of multiple stable conformers (with different relative orientations of the hydrogen bonding networks, or different coordination number between the Na^+^ and CD, for example) that are so structurally similar that they cannot be separated using our SLIM-IMS device; or (3) the monomodal ATD results from multiple conformers that rapidly interconvert.

Option (3) appears highly unlikely, given the rigid geometry of both sodiated β-CD and α-CD and the consequent significant strain associated with the concerted rearrangement of the hydrogen-bonded hydroxyl groups. We cannot completely rule out the possibility of (2), that structurally similar [α-CD + Na]^+^ or [β-CD + Na]^+^ conformers are present within the same ATD peak. Structural changes resulting from different coordination numbers of the sodium cation will likely lead to structural distortions easily resolved by our ion mobility device. Even if two or more cyclodextrin conformers would have such similar CCS that our SLIM-IMS is unable to resolve them, corresponding calculations would also not be able to reproduce the slight difference in CCS, as the resolution of our IMS instrument exceeds the accuracy of such calculations. As discussed more fully below, cryogenic IR spectroscopy is highly sensitive to structural differences, and the number of bands in each of the spectra of Fig. 4 is consistent with presence of single conformers. Thus, the most straightforward conclusion from the data of Fig. 4 is that both sodiated α- and β-CD exist as single conformers in the gas phase.

The cryogenic IR spectra corresponding to the monomodal ATD distributions of sodiated α- and β-CD shown in Fig. 4 were obtained by gating the monomer ATD peak, storing and tagging the corresponding ions in a cryogenic ion trap, and monitoring the depletion of tagged species as a function of IR wavenumber using a ToF spectrometer. Both IR spectra show similarities, with three distinct regions of IR transitions representing three types of OH oscillators. The lowest energy range of the spectra (3330–3450 cm^−1^) exhibits broad bands for both [α-CD + Na]^+^ and [β-CD + Na]^+^. The [α-CD + Na]^+^ spectrum presents a slightly asymmetric feature at 3375 cm^−1^, while the spectrum of [β-CD + Na]^+^ shows three distinct bands with maxima at 3355, 3395 and 3410 cm^−1^. Previous theoretical studies on neutral and metal-complexed CDs predict a tight hydrogen bonded network between the C6 hydroxyls on the narrow rim, and find that Na^+^ and other metal cations preferentially coordinate with these hydroxyls.^64–67^

**Fig. 5:**
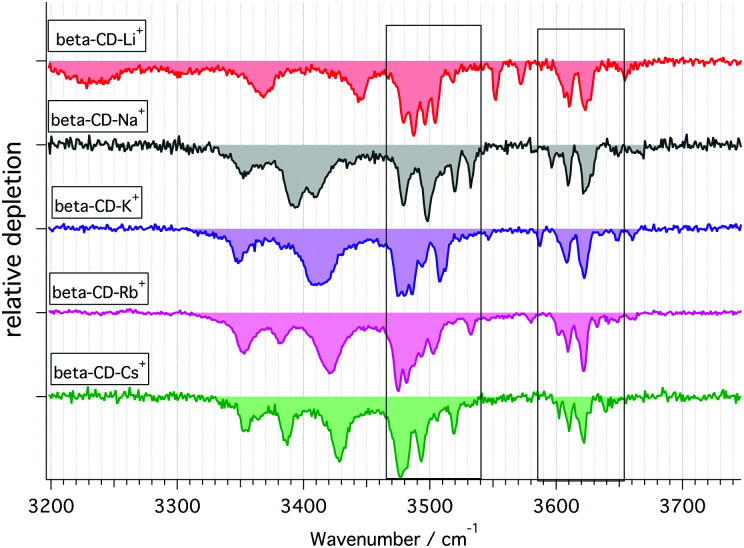
Cryogenic IR spectra of β-CD complexed with group I cations. The two marked areas denote the transitions associated to the C2 and C3 OH oscillators.

Additional cryogenic IR spectra of β-CD complexed to other group I metal cations (Fig. 5) demonstrate that the ionic radius strongly affects the bands below 3450 cm^−1^. The IR spectra in this wavenumber region show three major bands for all complexed β-CD cations, whereas only the complexes with Cs^+^ and Rb^+^ show significant spectral similarity. These experimental findings support the notion that the C6 hydroxyls are binding sites for the metal cations, but also hint to the formation of additional hydrogen bonding networks between these C6 OHs, which vary in number and strength depending on the complexed metal cation. For instance, the small Li^+^ will be complexed by a reduced amount of C6 O-atoms, thus likely promoting one or more strong hydrogen bonds between the hydroxyls undergoing metal complexation and other neighbouring free C6 OHs. Such a strong hydrogen bond could be the reason for the broad low energy band centred at 3225 cm^−1^, which is only present in the IR spectrum of [β-CD–Li]^+^. In contrast to the lithium case, the large radii Cs^+^ and Rb^+^ cations will be complexed by many of the seven C6 hydroxyl groups of β-CD, preventing the strong hydrogen bonds predicted for the Li^+^ complex. These symmetry considerations agree with the theoretical predictions of Gamez and coworkers, which find a trivalent coordination of the C6 hydroxyls for the [α-CD–Li]^+^ complex, whereas the lowest energy [α-CD–Cs]^+^ complex consists of five C6 Oxygens binding to the metal cation.^67^ Based on these arguments, an even stronger symmetric complexation could be inferred from the [α-CD–Na]^+^ spectrum (Fig. 4(a)), where the presence of one single transition centred at 3370 cm^−1^ plausibly arises from a structural arrangement with the C6 hydroxyls binding to the metal cation, leading to a network of concerted hydrogen bonds. In contrast, the low energy spectral region in sodiated β-CD hints to a more asymmetric binding pattern to the metal cation.

The differences between sodiated α- and β-CD in this spectral region can be thus attributed to different sodium binding patterns and consequent symmetry distortions of the two molecules. In summary, we assign these low energy transitions to the C6 hydroxyl stretch vibrations and associate the differences in this region to the number of metal-binding C6 hydroxyls as well as the resulting strength of additional intramolecular hydrogen bonds between these OHs.

The higher energy part of the spectra shows two groups of narrow transitions in the 3450–3550 cm^−1^ and 3560–3700 cm^−1^ ranges. Based on the above assignment, these oscillators correspond to the C2 and C3 hydroxyls located on the wider rim. Theoretical studies have repeatedly shown that these hydroxyls form a hydrogen-bonded network that can adopt two different configurations around the rim (cw and ccw), either with the C3 hydroxyls hydrogen-bonded to the partially free C2 hydroxyls of the adjacent glucose moieties, or the reversed configuration. While these two possible configurations prevent us from assigning the groups of IR transitions to specific hydroxyls, the 3450–3550 cm^−1^ and 3560–3700 cm^−1^ transitions correspond to the hydrogen-bonded and partially free hydroxyls on the wide rim, respectively. The similarity between sodiated α- and β-CD in these spectral regions further supports that the metal cation does not bind here, but rather at the narrow rim.

### Cryogenic IR spectra of CID fragments

**Fig. 6:**
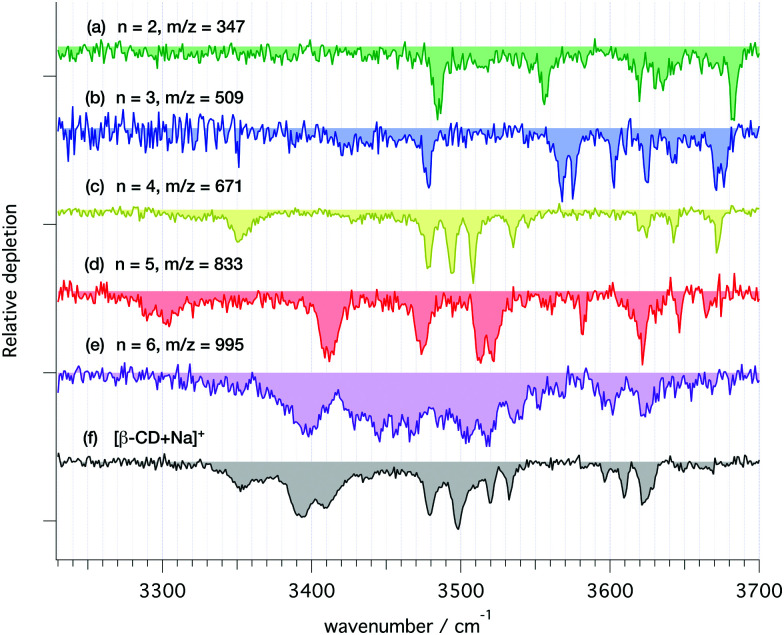
Cryogenic IR spectra of the main CID fragments of [β-CD + Na]^+^ (a–e) and the parent ion (f). The index *n* refers to the number of monosaccharides in each fragment.

Sodiated fragments of [β-CD + Na]^+^ were produced by collision-induced dissociation between the ion funnel (IF, Fig. 2) and the hexapole followed by *m*/*z* selection in the first quadrupole and storage in the cryogenic trap, where their IR spectra were recorded (Fig. 6(a–f)). These spectra provide well-resolved transitions that can be used to help determine the structure(s) of the fragment ions. Note that the IR spectrum of the precursor sodiated β-CD (Fig. 6(f)) obtained on the mass spectrometer shown in Fig. 2 is highly consistent with the [β-CD + Na]^+^ spectrum (Fig. 4(b)) obtained using the IMS-MS device (see Fig. S4, ESI†). This spectral comparison demonstrates that the conformational landscape of sodiated β-CD is similar in both instruments. Following all above arguments, we expect that the CID fragments of Fig. 3(a) are produced from a single [β-CD + Na]^+^ conformer.

To associate the observed spectroscopic features with a specific structure, compositional sampling simulations followed by electronic structure and vibrational frequency calculations were performed on the sodiated fragments containing 2, 3, and 4 residues (*m*/*z* 347, 509, and 671, respectively).

The spectra of the 2- and 3-residue sodiated fragments (Fig. 6(a and b)) exhibit a series of well-resolved bands above 3530 cm^−1^ as well as one band in the 3480–3490 cm^−1^ range. Neither spectrum show bands below 3470 cm^−1^. In both cases, the observed number of transitions match the expected number of OH stretch vibrations if one assumes that there is only a single conformer. The most distinct IR signatures are observed for the 4-residue fragment (Fig. 6(c)). The three clumps of well-resolved bands resemble the spectral features observed for [α-CD–Na]^+^ (see Fig. 4(a)). Synthesis of similar cyclic tetrasaccharides has been previously carried out,^68,69^ and very recently the first synthesis of a 4-member cyclodextrin was reported.^70^ However, both the significant strain imposed on the glucose units^71^ together with the predicted high barrier to re-cyclization make the formation of a 4-member cyclodextrin unlikely. Despite this, we tested this hypothesis computationally, as discussed below.

In contrast to the spectra of the smaller fragments, the 5- and 6-residue ions (Fig. 6(d and e)) exhibit a significant number of broad bands at lower wavenumber, which are characteristic of hydrogen-bonded OH stretch vibrations. The spectrum of the 5-residue fragment contains a few weak bands in the 3280–3320 cm^−1^ range, four strong broad features in the 3400–3550 cm^−1^ range, as well as several weaker transitions at higher wavenumber. There are fewer bands for this fragment than the number of OH oscillators, indicating that some bands overlap. The 6-residue fragment (Fig. 6(e)) exhibits a less resolved IR spectrum, with overlapping bands across the entire range between 3360–3630 cm^−1^. Comparing the *n* = 6 fragment to the isomeric [α-CD–Na]^+^ (Fig. 4(a)) reveals a difference in the number, position, and breadth of the bands, which may reflect the difference between a more symmetric, rigid, cyclic structure and an open, asymmetric one. The spectroscopic differences between the *n* = 6 fragment and [α-CD + Na]^+^ thus imply the presence of a substantial energy barrier to re-cyclization of the CID fragment. Rather, one (or several) open fragment structure(s) must be stabilized and kinetically trapped in its (their) potential minimum (minima) through collisional cooling.

### Comparison with theory

**Scheme 1:**
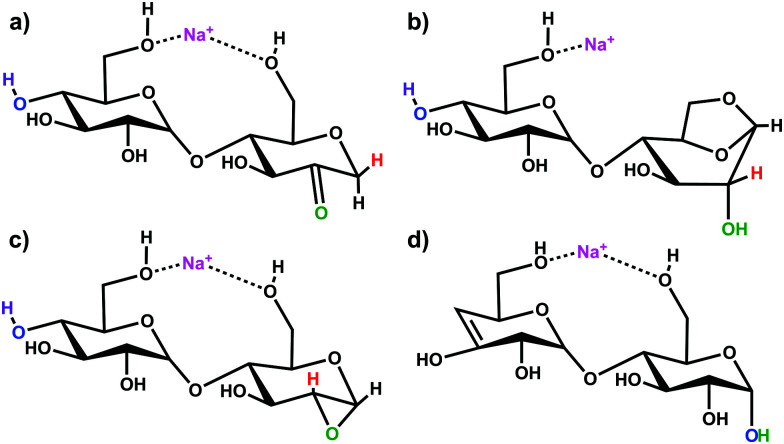
Putative ion structure types potentially formed from [β-CD + Na]^+^, illustrated for the 2-residue, *m*/*z* 347 species: (a) 2-ketone B_2_; (b) 1,6-anhydro B_2_; (c) 1,2-anhydro B_2_; and (d) Z_2_ ion.

Simulations of the 2-, 3-, and 4-residue fragments resulted in a significant number of low energy conformations. For the 2- and 3-residue fragments, simulations predict four distinct types of energetically stable structures (Scheme 1), with an additional cyclic form found for the 4-residue dissociation product.

Three of the four fragments are B ions, while the remaining fragment species is a product of a Z-type fragmentation. Our B3LYP data support 2-ketone B-type fragments^43,44,72,73^ as the lowest energy structures. The mechanistically less likely to be formed 1,6-anhydro B ion structure^45,74^ is next followed by the epoxide 1,2-andydro structures, which are consistently least energetically favourable (≥85 kJ mol^−1^). The Z fragments are predicted to be 10–20 kJ mol^−1^ higher in energy than the 2-ketone B ion structures. Large basis set M06-2X and ωb97X-D single-point calculations predict the 2-ketone and 1,6-anhydro terminated B ion structures and the Z ion structures to be of similar relative energy. All levels of theory substantially disfavour the strained 1,2-anhydro terminated B ion structures. The lowest energy structures of each type of fragment are shown in Fig. S5–S7 of the ESI,† and the corresponding relative energies are indicated in Table S1 (ESI†).

**Fig. 7:**
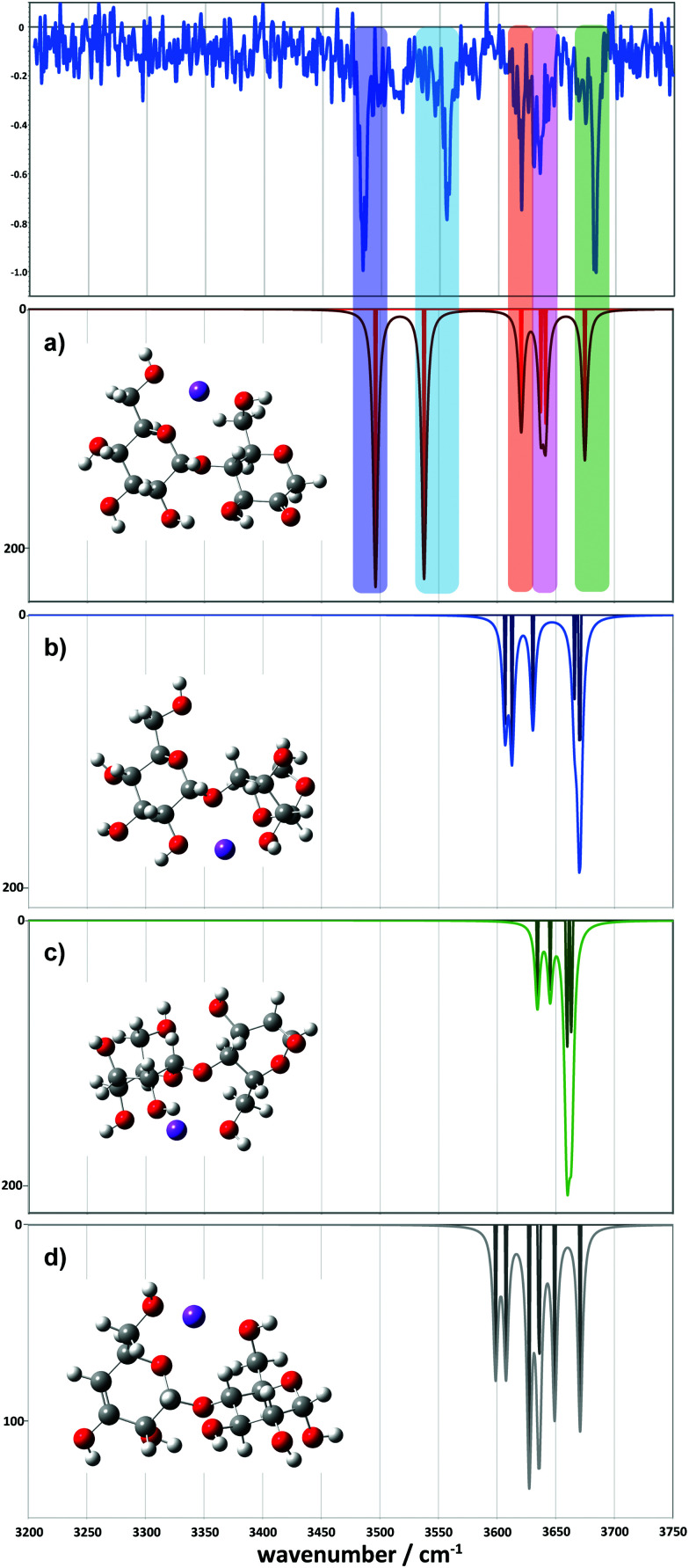
Comparison between the experimental (top) and simulated IR spectra for the *m*/*z* 347 fragment. (a) Represents the ketone, (b) the 1-6 anhydro, (c) the 1-2 anhydro and (d) the Z fragment structures.

For the 2-residue fragment (*m*/*z* 347), Fig. 7 depicts the comparison between the experimental spectrum and the IR transitions calculated for the lowest energy B_2_ and Z_2_ ion structures. This result strongly suggests formation of a 2-ketone B_2_ ion structure (Fig. 6a). Consequently, we assign: (1) the ∼3485 cm^−1^ band to the symmetric O–H stretch of the carbon 3 hydroxyl of residue 2, H-bonded to the adjacent ketone oxygen and the O–H of the carbon 2 hydroxyl of residue 1 (also H-bonded); (2) the 3556 cm^−1^ band to the asymmetric stretch of the same hydroxyl groups; (3) the 3620 cm^−1^ band to the carbon 4 hydroxyl of residue 1, O–H stretch; (4) the 3630 cm^−1^ band to the carbon 3 hydroxyl of residue 1, O–H stretch; (5) the 3636 cm^−1^ band to the carbon 6 primary hydroxyl of residue 2, O–H stretch; and (6) the 3683 cm^−1^ band to the carbon 6 primary hydroxyl of residue 1, free O–H stretch. Following these assignments, and in contrast to the cyclic structures proposed for the metal complexes (Fig. 5), the low energy bands observed for the sodiated ring-opened β-CD fragments do not necessarily relate to the C6 hydroxyls.

**Scheme 2:**
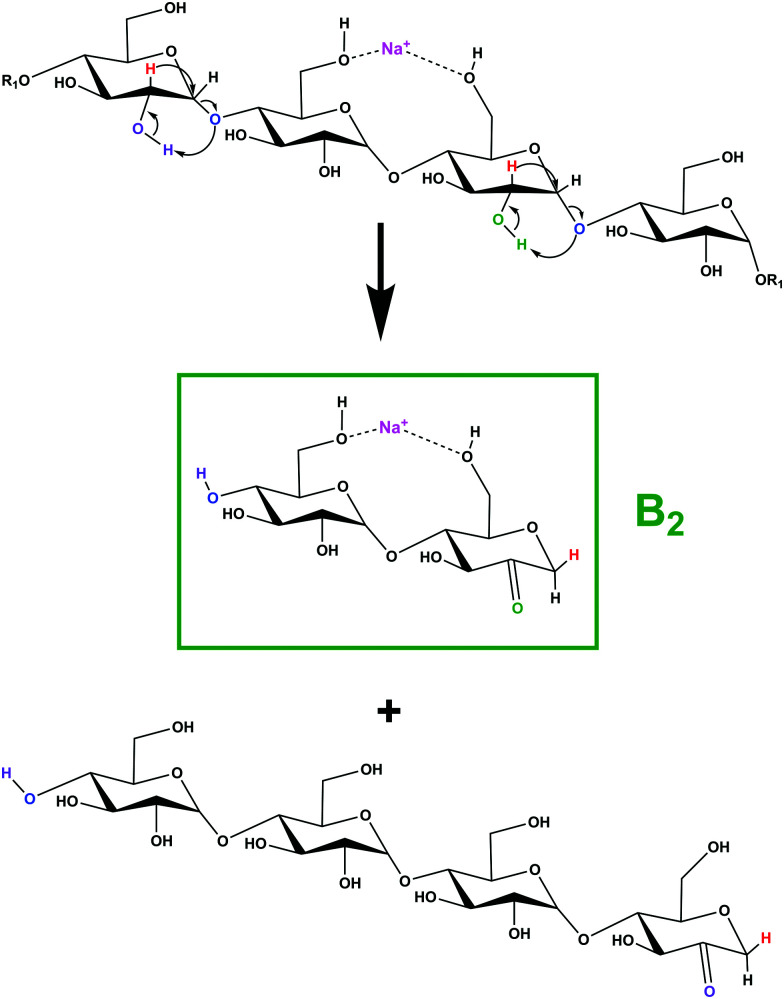
Proposed fragmentation mechanism for the formation of the *m*/*z* 347 fragment.

The proposed mechanism for this fragmentation process is presented in Scheme 2 and is adapted from earlier proposals for linear systems.^43,44,72,73^ Other candidate fragmentation pathways are detailed in Fig. S8 (ESI†). The mechanism involves abstraction of a carbon 2 hydroxyl proton, formation of a ketone, and a 1,2-hydride shift with concerted glycosidic bond cleavage.^43,44,72,73^ This ring opening simultaneously creates the first 2-ketone and a new C4 hydroxyl group from the cleaved glycosidic linkage, *i.e.*, nominally a linear B_7_ 2-ketone terminated structure. Further dissociation of a second glycosidic bond in an identical manner results in generation of 2-residue and 5-residue structures with the sodium cation in an ion–molecule complex.^74^ Provided the complex has sufficient lifetime, a competition for the sodium cation ensues in which the fragment with the larger sodium affinity should most frequently predominate.^45,74^ If the 2-residue fragment separates with the sodium attached, an *m*/*z* 347 ion is detected, and if the 5-residue fragment separates with the sodium attached, an *m*/*z* 833 ion is detected.

**Fig. 8:**
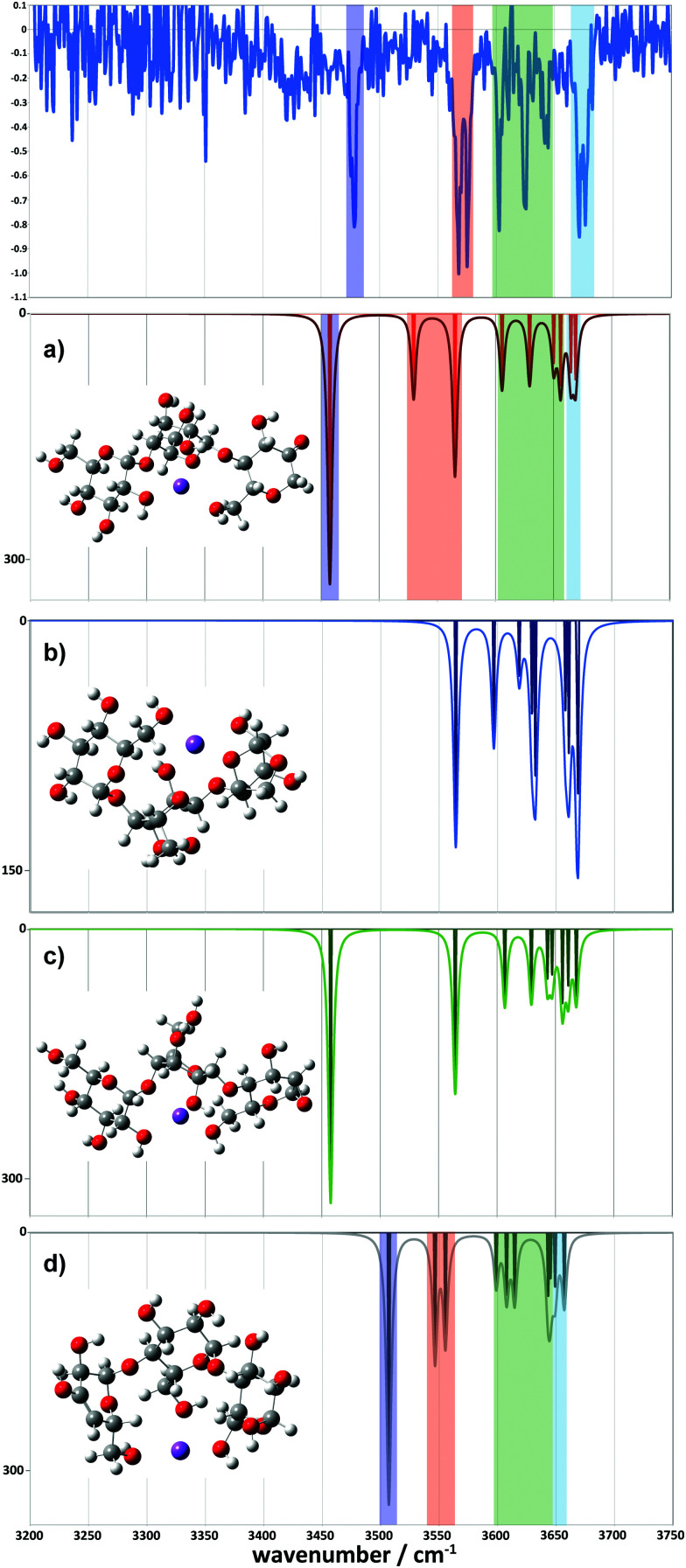
Comparison between the experimental (top) and simulated IR spectra for the *m*/*z* 509 fragment. (a) represents the ketone, (b) the 1-6 anhydro, (c) the 1-2 anhydro B_3_ and (d) the Z_3_ fragment structures.

Comparison between experimental and theoretical IR spectra of the 3-residue fragment is less conclusive (Fig. 8). None of the theoretical spectra of the lowest energy structures are perfectly consistent with experiment. The lowest energy 2-ketone type structure is reasonably consistent with experiment (Fig. 8a, mean deviation = 5.9 cm^−1^, *R*^2^ = 0.97). However, the Z_3_-type structure shows a similar quality of fit (Fig. 8d, mean deviation = 12.0 cm^−1^, *R*^2^ = 0.94). In contrast, the 1,2-anhydro B_3_ prediction lacks sufficient intermediate energy bands and there is no evidence to support any population of the 1,6-anhydro B_3_ structure. The latter is consistent with structural arguments against the feasibility of the cross-ring proton transfers necessary to generate these structures from alpha linked sugars (Fig. S8c, ESI†).

If our tentative assignment of the 2-ketone B_3_ or the Z_3_ ion structure for the *m*/*z* 509 peak is correct, this offers a potential explanation for the origin of the *m*/*z* 671 spectrum—that is, analogous 3-residue and 4-residue structures together with a sodium cation comprise the ion–molecule complex formed following dissociation of the 2 glycosidic bonds. As both neutrals are of similar size and comprise the same functional groups, similar sodium affinities should be expected, enabling both ions to be formed and detected, provided the ion–molecule complex has sufficient lifetime to enable sodium cation transfer between the fragments.^45,74^

Following this argument, either a 2-ketone B_4_ or a Z_4_ structure would be expected for the 4-residue fragment, but none of the lowest energy 2-ketone B_4_ nor Z_4_ theoretical spectra are particularly consistent with the experimental spectrum for *m*/*z* 671 (Fig. S7, ESI†). This is similarly true for the other fragment structure types including potential cyclic candidate structures.

**Fig. 9:**
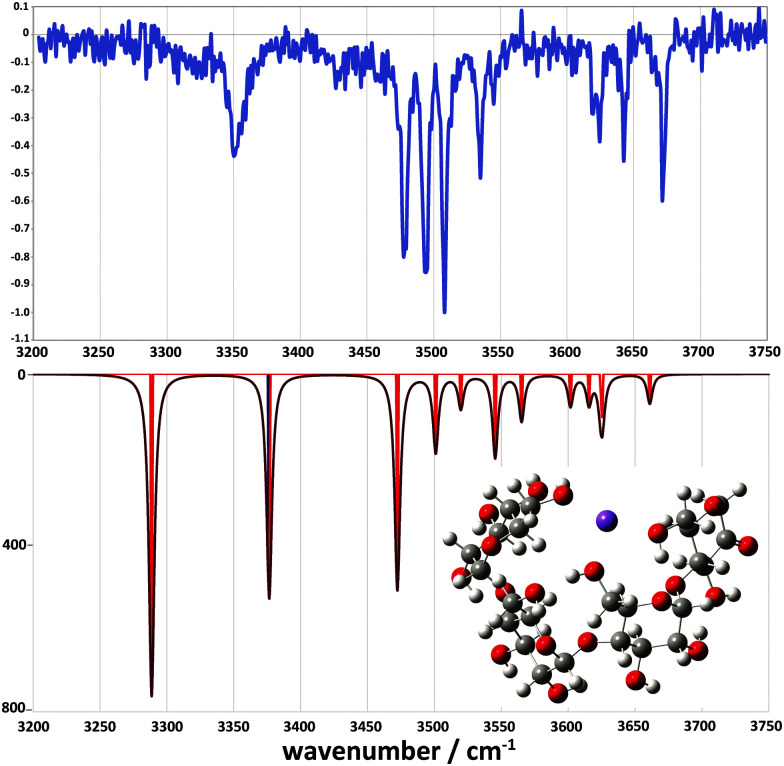
Comparison between the experimental (top) and simulated IR spectra for the *m*/*z* 671 fragment. The simulated spectrum represents a 2-ketone B_4_ structure at 7.9 kJ mol^−1^.

However, we did locate a low energy 2-ketone B_4_ structure that was far more consistent with the experimental spectrum (Fig. 9). The broad bands at low wavenumber correspond to strongly H-bonded C6–OH stretches (predicted at 3290 and 3377 cm^−1^), consistent with the broad feature at ∼3310–3380 cm^−1^. The bands at intermediate wavenumber correspond to a mixture of H-bonded environments (C3–OH, C6–OH, C2–OH, C2–OH, C2–OH) at 3473, 3502, 3520, 3546, and 3566 cm^−1^, respectively. The bands at high wavenumber (3602, 3616, 3626, and 3662 cm^−1^) comprise three C3–OH stretches and a free C6–OH stretch.

## Conclusions

This work reports fingerprint vibrational spectra for a series of metal complexed β-CD cations, focusing on sodiated β-CD and its CID products. We obtain distinct, well-resolved infrared spectra for each of the main fragments. Simulations followed by electronic structure and vibrational frequency calculations were performed for the 2-, 3-, and 4-residue ions corresponding to B or Z ion structures and compared to the experimental results. The main findings of this work are:

(1) Collisional activation of [β-CD + Na]^+^ results in a series of fragment ions differing by 162 u. Cryogenic IR spectroscopy experiments on the major fragment ions provide well-resolved vibrational fingerprints in the 3200–3750 cm^−1^ spectral region.

(2) Our combined experiments and calculations support the formation of a single gas-phase conformer in each of the *m*/*z* 347, 509, and 671 species. The experimental spectrum for *n* = 5 suggests formation of a single fragment conformer, while that for the 6-residue fragment suggests the presence of more than one stable conformer.

(3) There are four main types of potential fragmentation products for the 2-, 3-, and 4-residue fragments. For the 2-residue fragment, the predicted lowest energy 2-ketone-type fragment is clearly the most similar to the experimental spectrum. For the 3-residue fragment, both the predicted lowest energy 2-ketone-type and Z_3_-type fragments show significant similarities to the experimental spectrum. The 4-residue fragment is also most consistent with a 2-ketone type structure, which in turn would support the same for the 3-residue fragment (Scheme 2). Thus, all the fragments seem to form B_*n*_ 2-ketone structures.

(4) The results presented here also demonstrate that cryogenic IR spectroscopy and theory can be used to directly relate spectroscopic patterns in CD–metal cationic complexes to the location and degree of coordination between the host CD and the metal ion.

In a broader context, this work demonstrates the potential of our method to unravel atomic-level structural features of complex biomolecular systems that can help address key macromolecular functions.

## Author contributions

E. C. conceived the experiment; J. M. R. and B. J. B. performed the theoretical simulations; J. M. R. and B. J. B. analysed the simulation results; R. P. P. and E. C. carried out the experiments. R. P. P. analysed the experimental data; A. B. K. took the data shown in Fig. 4; J. M. R., A. B. K., B. J. B., and T. R. R. contributed to data discussion and interpretation; R. P. P., J. M. R., B. J. B., T. R. R. and E. C. wrote the manuscript.

## Conflicts of interest

There are no conflicts to declare.

## Supplementary Material

CP-023-D1CP01058A-s001

CP-023-D1CP01058A-s002

CP-023-D1CP01058A-s003

CP-023-D1CP01058A-s004

CP-023-D1CP01058A-s005
