# Supplementary material for: Unravelling the structures of sodiated β-cyclodextrin and its fragments
Source: Phys Chem Chem Phys. 2021 Jun 7;23(24):13714–23. doi: 10.1039/d1cp01058a (PMC8220536; doi:10.1039/d1cp01058a)
Supplement: CP-023-D1CP01058A-s001 [file CP-023-D1CP01058A-s001.pdf]

Electronic Supplementary Material (ESI) for Physical Chemistry Chemical Physics.
This journal is © the Owner Societies 2021

SUPPORTING INFORMATION

Unravelling the structures of sodiated β-cyclodextrin and its fragments

Jordan M. Rabus,[b†] Robert P. Pellegrinelli,[a†] Ali Hassan Abi Khodr,[a] Benjamin J. Bythell,[b] Thomas R. Rizzo[a] and Eduardo Carrascosa,*[a†]

[a] Laboratoire de Chimie Physique Moléculaire, École Polytechnique Fédérale de Lausanne, EPFL SB ISIC LCPM, Station 6, CH-1015 Lausanne, Switzerland

[b] Department of Chemistry and Biochemistry, Ohio University, 391 Clippinger Laboratories, Athens, Ohio 45701, United States

Table of Contents

Cover page S1

MS and MS^2 spectra of sodiated β-CD in a commercial QTof spectrometer and resulting relative fragment intensities S2

Schematic representation of the IMS instrument and comparison of IR spectra S3

Comparison between Experiment and Theory based on lowest energy structures S4-S6

Proposed fragmentation mechanisms for CID products S7

Relative energies of candidate fragments calculated at different density functionals S8

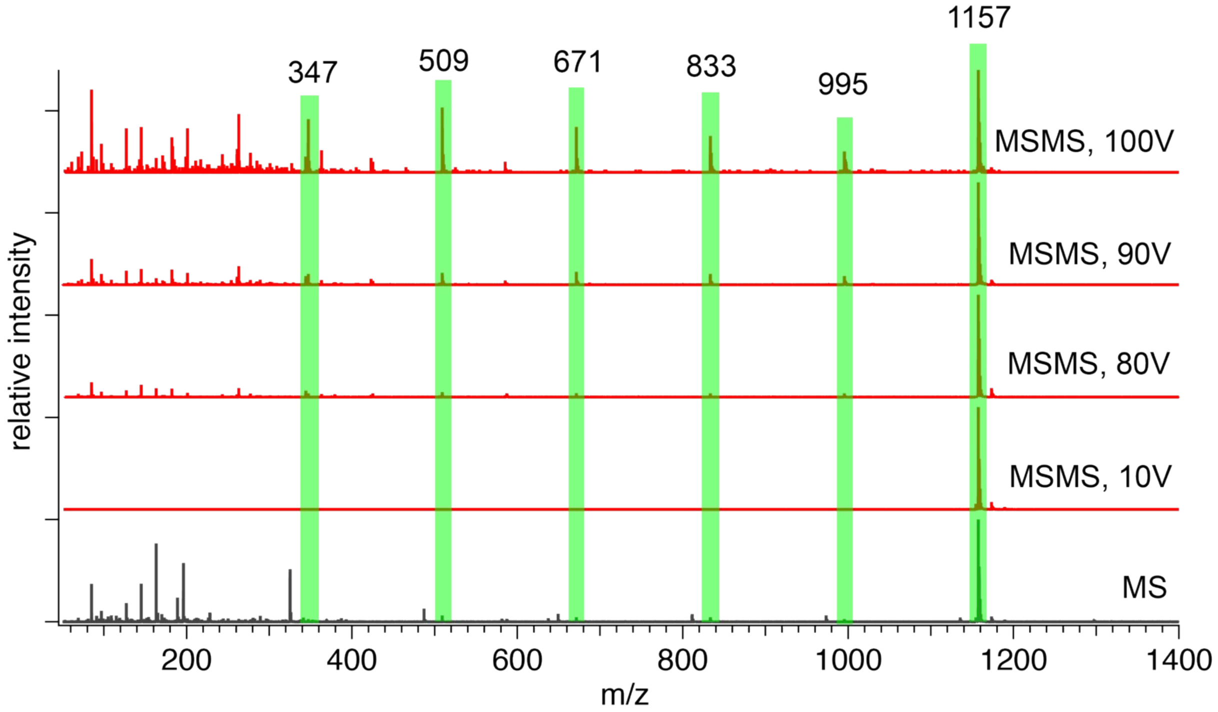

Figure S1: MS and MS^2 spectra of sodiated β-CD at different fragmentation energies in the collision cell of a commercial QTof spectrometer (Waters Permier). The MS spectrum shows the presence of protonated fragments either already present in solution or produced *in-source*. Formation of the main sodiated fragments starts at voltage difference of around 80V between the quadrupole bias and the collision cell bias. The main fragment masses are marked by green shaded areas.

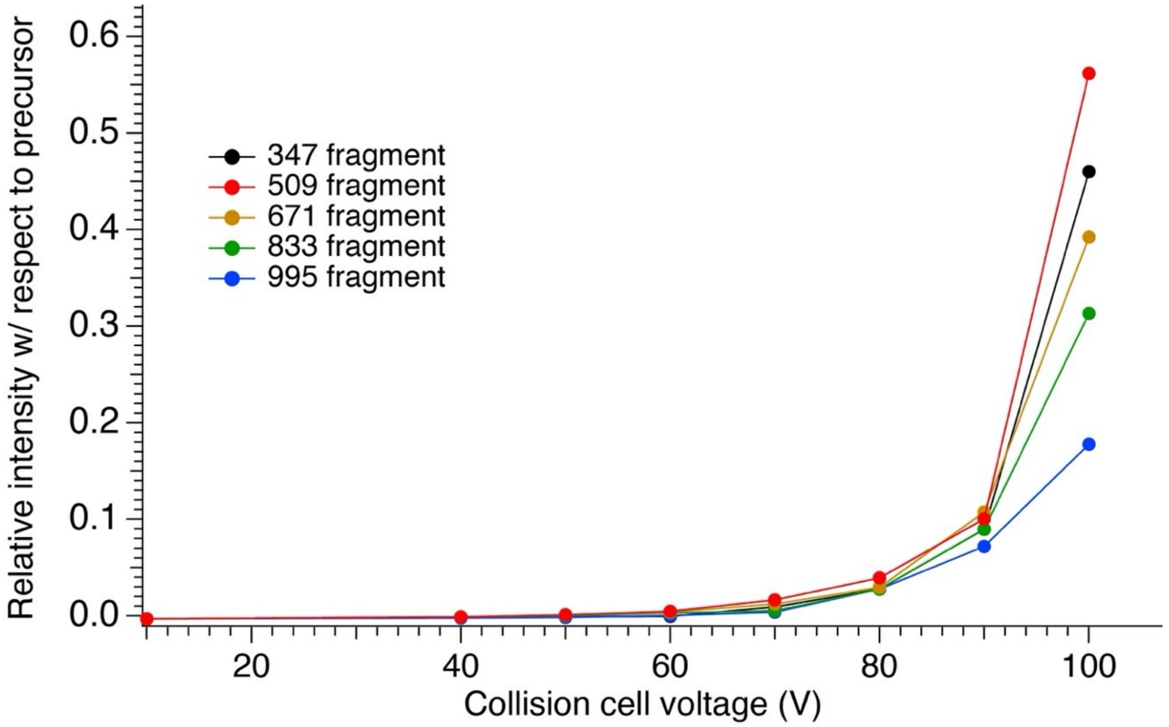

Figure S2: Relative intensity of CID fragments of sodiated β-CD normalized to the precursor intensity as a function of collision cell energies on a commercial QTof spectrometer (Waters Permier).

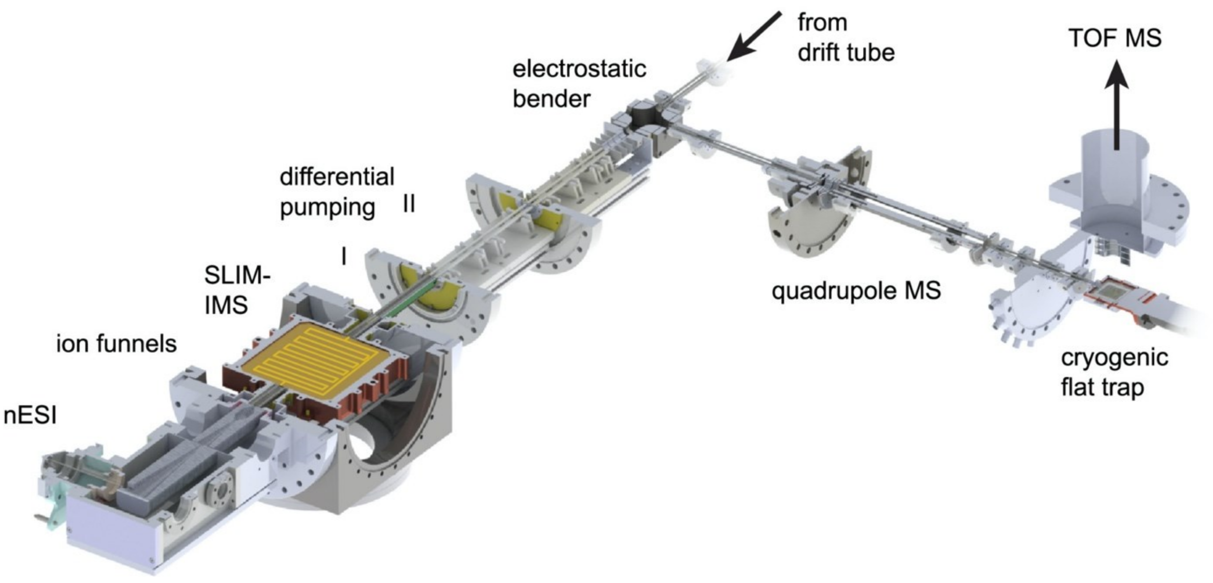

Figure S3: Schematic representation of the homebuilt ion mobility mass spectrometer used to obtain the ATDs and cryogenic spectra shown in Figure 4 of the manuscript, adapted with permission from reference 38. The apparatus consists of the same electrospray source, an ion funnel trap, a homebuilt SLIM type ion mobility spectrometer, a quadrupole mass filter and a cryogenically cooled ion trap coupled to an orthogonal time-of-flight spectrometer.

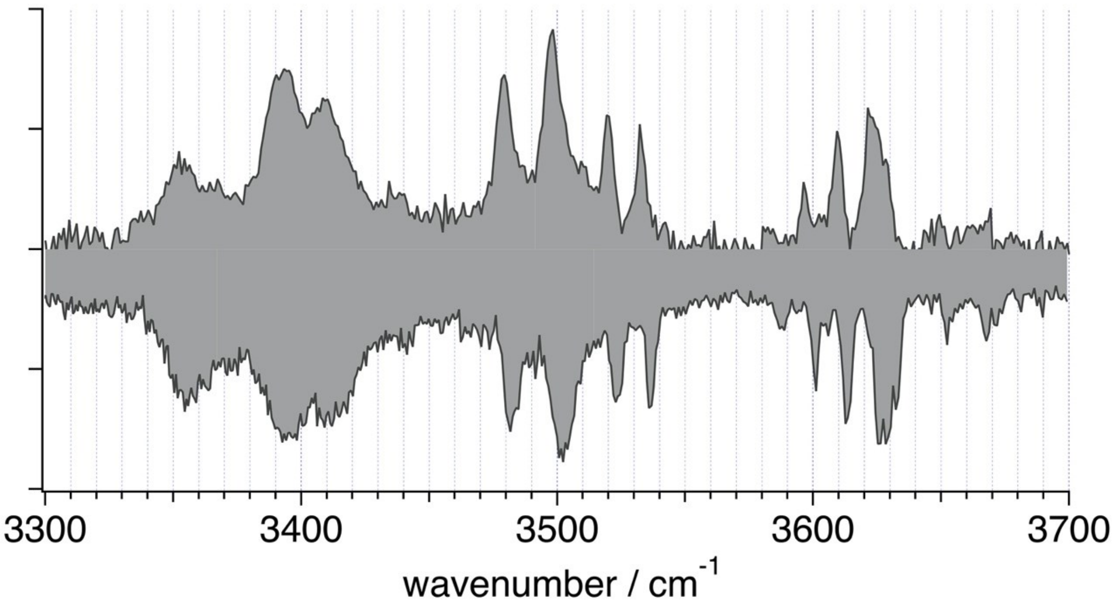

Figure S4: Cryogenic IR spectra of sodiated β-CD measured on the tandem mass spectrometer shown in Figure 2 of the manuscript (upper spectrum) and on the IMS-MS instrument shown in Figure S3 (lower spectrum). Both spectra were taken at the same temperature conditions and using N_2 as tagging molecules. The small shift observed in the bands come from a slightly different laser calibration.

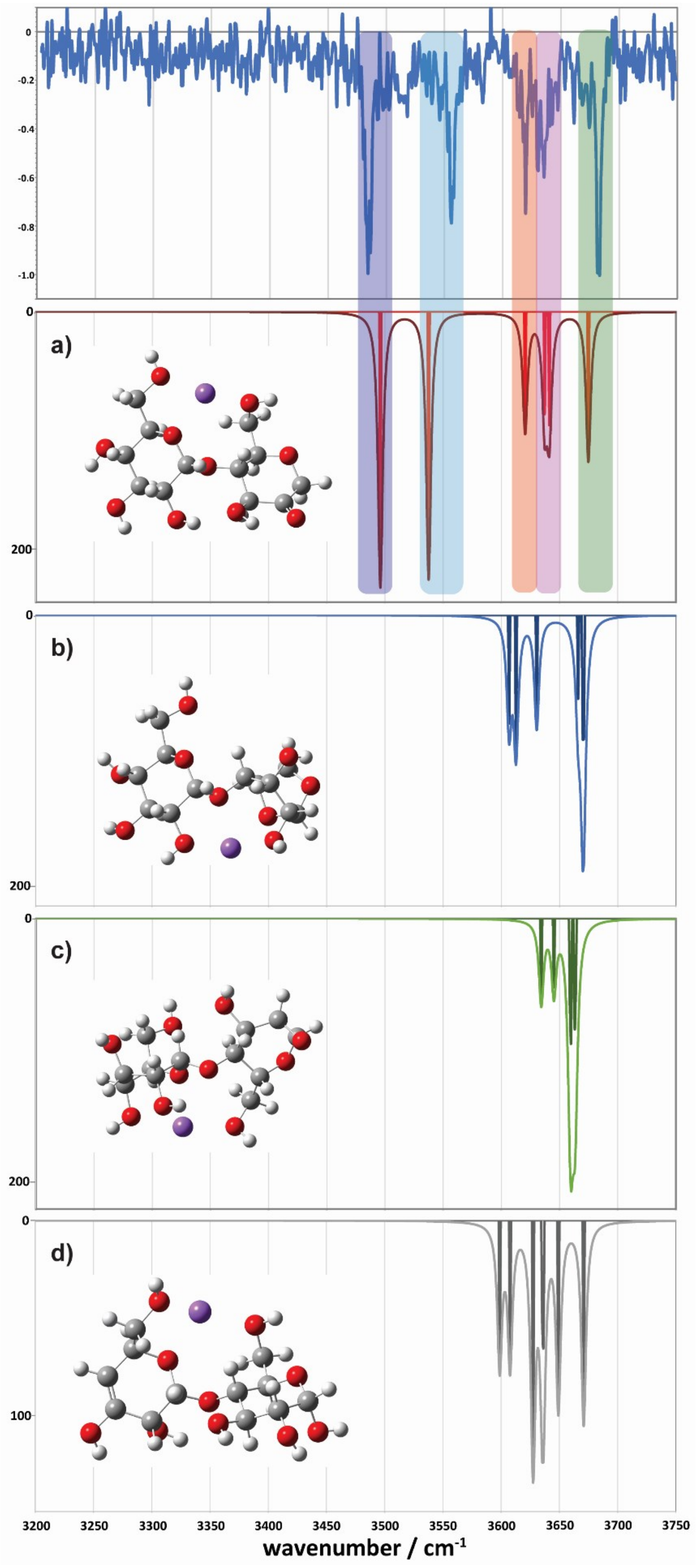

Figure S5: Comparison of *m/z* 347 lowest energy theoretical spectra with the experimental IR spectrum: lowest energy (a) 2-ketone B_2 ion structure; (b) 1,6-anhydro B_2 ion structure; (c) 1,2-anhydro B_2 ion structure; (d) Z_2 ion structure calculated at the B3LYP/6-31+G(d,p) level of theory. This Figure is reproduced from Figure 7 of the manuscript as single-column, full size figure for better visibility of the results.

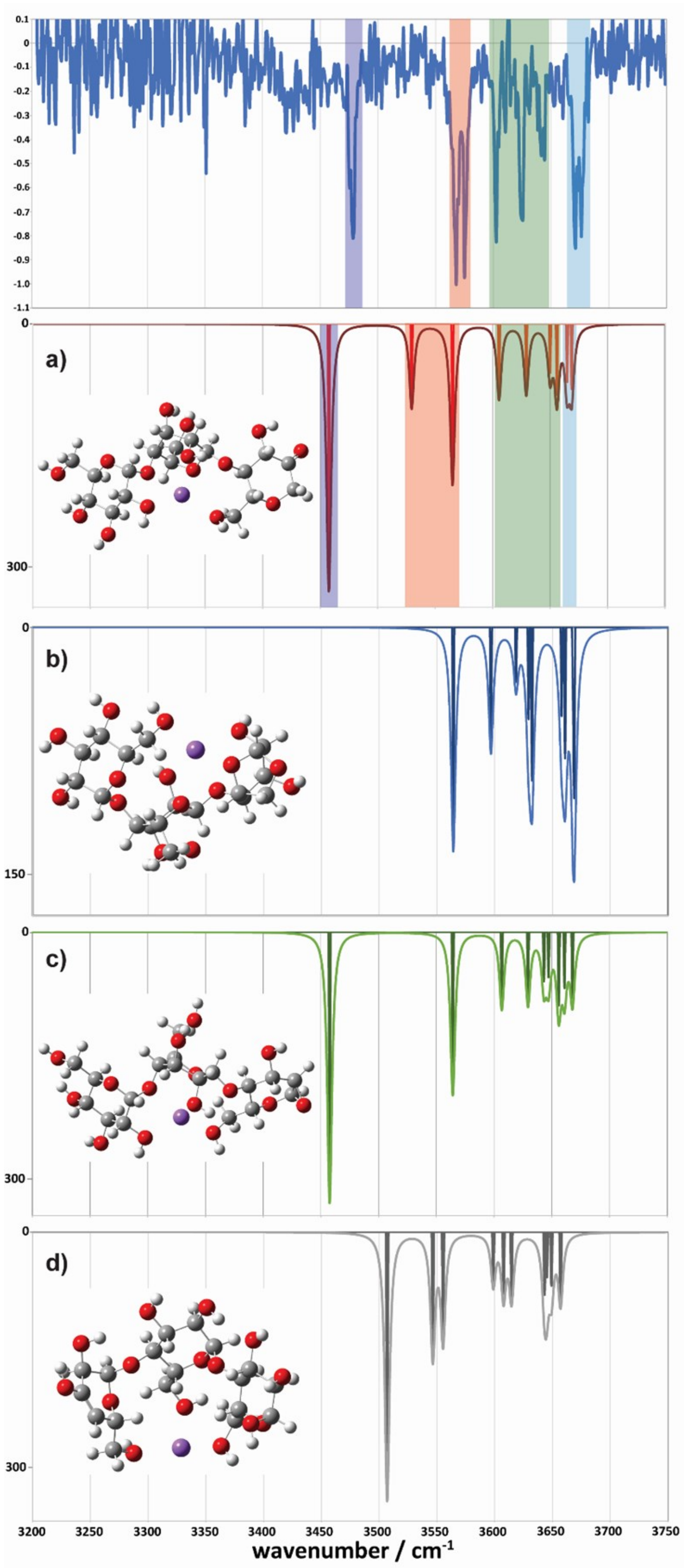

Figure S6: Comparison of *m/z* 509 lowest energy theoretical spectra with the experimental IR spectrum: lowest energy (a) 2-ketone B_3 ion structure; (b) 1,6-anhydro B_3 ion structure; (c) 1,2-anhydro B_3 ion structure; (d) Z_3 ion structure calculated at the B3LYP/6-31+G(d,p) level of theory. This Figure is reproduced from Figure 8 of the manuscript as single-column, full size figure for better visibility of the results.

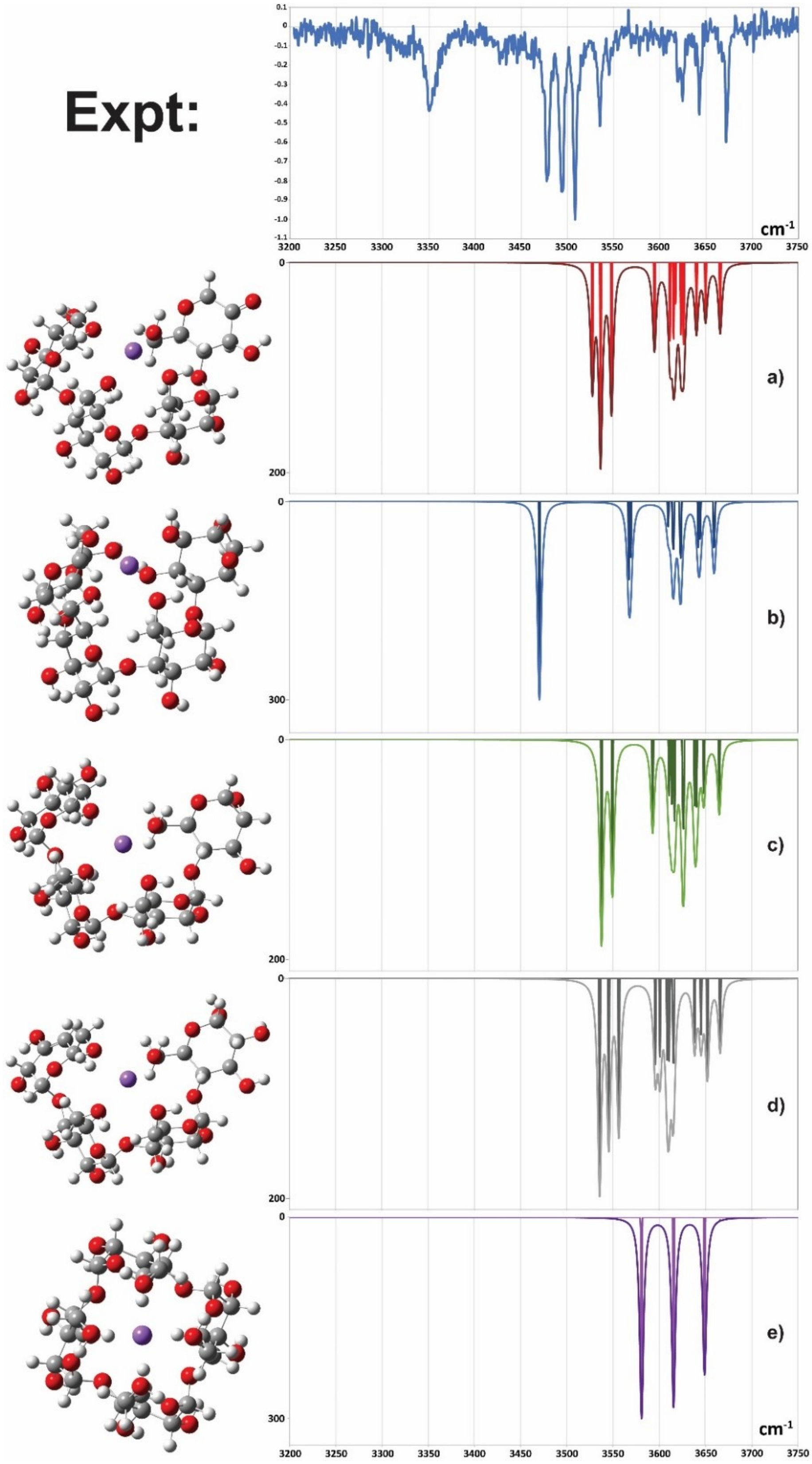

Figure S7: Comparison of *m/z* 671 lowest energy theoretical spectra with the experimental IR spectrum: lowest energy (a) 2-ketone B_4 ion structure; (b) 1,6-anhydro B_4 ion structure; (c) 1,2-anhydro B_4 ion structure; (d) Z_4 ion structure; (e) Cyclic ion (symmetrical) calculated at the B3LYP/6-31+G(d,p) level of theory.

Putative fragmentation mechanisms for CID products

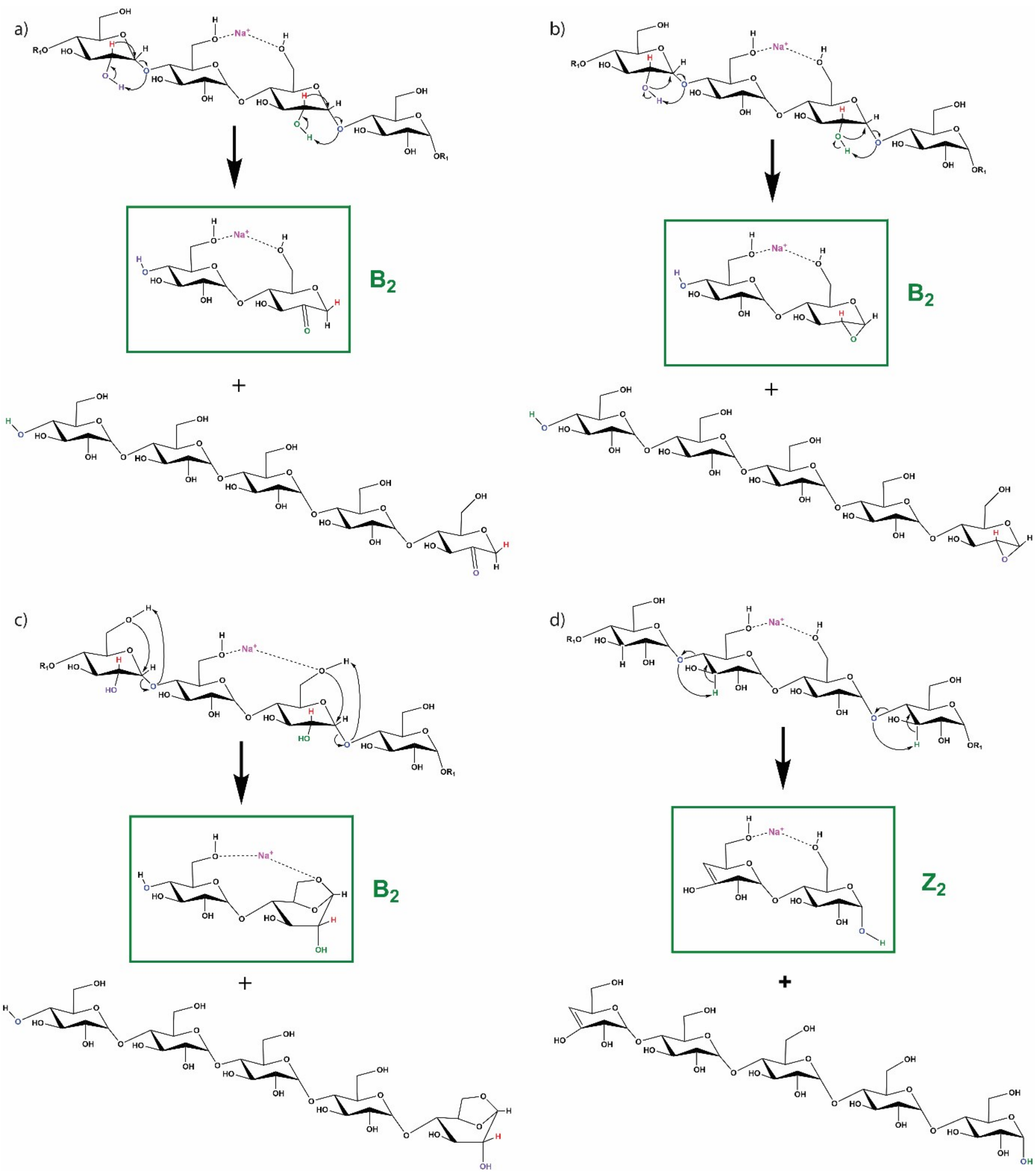

Figure S8: Simplified mechanisms for glycosidic bond dissociation in [β-CD+Na]$^+$ leading to the (a) 2-ketone B_2 ion, (b) the 1,2-anhydro B_2 ion, (c) the 1,6-anhydro B_2 ion products and the (d) Z_2 ion product. The latter involves formation of an alkene bond between C3 and C4 of the non-reducing end of the product ion.

Relative energies of the candidate ion structures calculated at different levels of theory

m/z 347 Structures	$\Delta E_{el,0K+ZPE}$ (ΔG_{298K}) B3LYP/6-31+G(d,p)	$\Delta E_{el,0K}$ M06-2X/6-311+G(2d,p)[a]	$\Delta E_{el,0K}$ wB97XD/6-311+G(2d,p)[a]
Ketone B_2	0 (0)	7.0	0
Z_2	14.9 (12.8)	14.4	15.8
1,6-andydro B_2	22.2 (24.2)	0	3.4
1,2-andydro B_2	106.4 (106.2)	84.8	90.0

Table S1. Relative energies of *m/z* 347 minima. Superscript "a" indicates single point calculations performed using the structure of each type calculated at the B3LYP/6-31+G(d,p) level of theory. Energies are given in kJ mol^{-1}.

m/z 509 Structures	$\Delta E_{el,0K+ZPE}$ (ΔG_{298K}) B3LYP/6-31+G(d,p)	$\Delta E_{el,0K}$ M06-2X/6-311+G(2d,p)[a]	$\Delta E_{el,0K}$ wB97XD/6-311+G(2d,p)[a]
Ketone B_3	0 (0)	9.9	0
Z_3	21.8 (32.0)	21.3	19.5
1,6-andydro B_3	29.2 (44.8)	0	0.3
1,2-andydro B_3	86.5 (92.0)	83.7	85.5

Table S2. Relative energies of *m/z* 509 minima. Superscript "a" indicates single point calculations performed using the structure calculated at the B3LYP/6-31+G(d,p) level of theory. Energies are given in kJ mol^{-1}.

m/z 671 Structures	$\Delta E_{el,0K+ZPE}$ (ΔG_{298K}) B3LYP/6-31+G(d,p)	$\Delta E_{el,0K}$ M06-2X/6-311+G(2d,p)[a]	$\Delta E_{el,0K}$ wB97XD/6-311+G(2d,p)[a]
Ketone B_4	0 (0)	0	0
Z_4	11.5 (14.9)	2.0	11.0
1,6-andydro B_4	41.6 (37.5)	1.4	4.2
Cyclic	56.3 (73.4)	58.6	10.2
1,2-andydro B_4	89.0 (90.2)	74.4	83.4

Table S3. Relative energies of *m/z* 671 minima. Superscript "a" indicates single point calculations performed using the structure calculated at the B3LYP/6-31+G(d,p) level of theory. Energies are given in kJ mol^{-1}.
